# Chromosomal-level genome and multi-omics dataset of *Pueraria lobata* var. *thomsonii* provide new insights into legume family and the isoflavone and puerarin biosynthesis pathways

**DOI:** 10.1093/hr/uhab035

**Published:** 2022-01-19

**Authors:** Xiaohong Shang, Xinxin Yi, Liang Xiao, Yansheng Zhang, Ding Huang, Zhengbao Xia, Kunpeng Ou, Ruhong Ming, Wendan Zeng, Dongqing Wu, Sheng Cao, Liuyin Lu, Huabing Yan

**Affiliations:** 1 Cash Crops Research Institute, Guangxi Academy of Agricultural Sciences, Nanning, Guangxi, 530007, China; 2 Wuhan Frasergen Bioinformatics Co., Ltd, Wuhan, Hubei, 430075, China; 3 Shanghai Key Laboratory of Bio-Energy Crops, Research Center for Natural Products, Plant Science Center, School of Life Sciences, Shanghai University, Shanghai, 200444, China; 4 College of Pharmacy, Guangxi University of Chinese Medicine, Nanning, Guangxi, 530200, China

## Abstract

*Pueraria lobata* var. *thomsonii* (hereinafter abbreviated as *Podalirius thomsonii*), a member of the legume family, is one of the important traditional Chinese herbal medicines, and its puerarin extract is widely used in the health and pharmaceutical industry. Here, we assembled a high-quality genome of *P. thomsonii* using long-read single-molecule sequencing and Hi-C technologies. The genome assembly is ~1.37 Gb in size and consists of 5145 contigs with a contig N50 of 593.70 kb, further clustered into 11 pseudochromosomes. Genome structural annotation resulted in ~869.33 Mb (~62.70% of the genome) repeat regions and 45 270 protein-coding genes. Genome evolution analysis revealed that *P. thomsonii* is most closely related to soybean and underwent two ancient whole-genome duplication events; one was in the common ancestor shared by legume species and the other occurred independently at around 7.2 million years ago, after its speciation. A total of 2373 gene families were found to be unique in *P. thomsonii* compared with five other legume species. Genes and metabolites related to puerarin content in tuberous tissues were characterized. A total of 572 genes that were upregulated in the puerarin biosynthesis pathway were identified, and 235 candidate genes were further enriched by omics data. Furthermore, we identified six 8-C-glucosyltransferase (*8-C-GT*) candidate genes significantly involved in puerarin metabolism. Our study filled a key genomic gap in the legume family, and provided valuable multi-omic resources for the genetic improvement of *P. thomsonii*.

## Introduction

*Pueraria lobata* (2*n* = 2*x* = 22) is a semiwoody, perennial liana that belongs to the Leguminosae family and is widely distributed throughout Asia, including China, Japan, Korea, and other regions in Southeast Asia, as well as in North and South America. As an economic crop, it contains puerarin and other functional components and is used in the production of both pharmaceuticals and health foods. The roots of both *P. lobata* (hereinafter abbreviated as *P. lobata*) and *P. lobata* var. *thomsonii* (Benth.) (hereinafter abbreviated as *Podalirius thomsonii*) have long been used for treating fever, toxicosis, indigestion, and liver damage from alcohol abuse in traditional Chinese medicine [1], which was recorded in *The Divine Husbandman’s Classic of Materia Medica* (*Shen Nong Ben Cao Jing*) compiled in the Eastern Han Dynasty (25–250 AD) [2]. Since *P. thomsonii* has much higher starch content and milder therapeutic effects, its root extract or powder is commonly consumed as a dietary supplement or an ingredient in sweet or savory dishes in Asian countries [3].

The classification of genus *Pueraria* is still largely in dispute. There are currently 20 accepted species, with synonyms crossing over in other genera, such as *Dolichos* and *Glycine* [4]. *P. lobata* var. *thomsonii*, sometimes called Thomson’s kudzu, or fenge, was recognized as one species in the *Pueraria* genus, along with *P. lobata* var. *montana* (hereinafter abbreviated as *P. montana*) and *P. lobata*. Synonyms such as *P. thomsonii* Benth., *P. lobata* var. *thomsonii* (Benth.) Maesen and *P. lobata* var. *chinensis* Ohwi have been used in various mentions [5]. *P. montana* is morphologically and genetically distinct from *lobata* and *thomsonii* [3]. Genetic markers, such as RAPD (random amplified polymorphic DNA), SSR (simple sequence repeat), and SNP (single nucleus polymorphism) markers, and sRNA probes have been used to characterize genetic variations in the species *P. thomsonii* and its close relatives [6–9]*.* However, most of the basic molecular and genetic analyses remain inconclusive in *Pueraria* due to the lack of genomic information. High-quality genome assemblies and their annotation in *P. thomsonii*would greatly improve the value of the resources available to distinguish and understand the genomic compositions.

**Figure 1:**
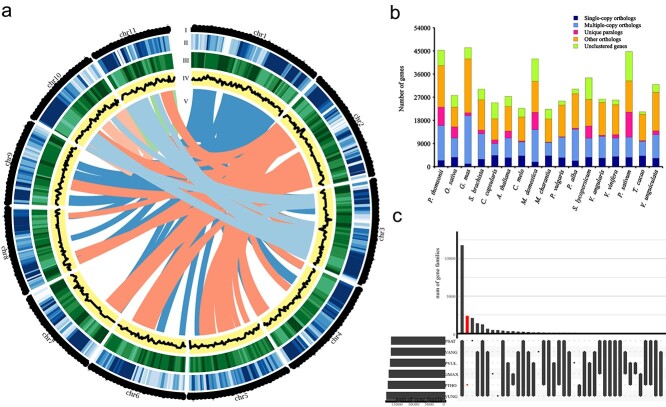
Genome landscape of assembly, annotation, and gene family clusters of *P. thomsonii*. **a** Overview of the *P. thomsonii* genomic features. Track I is the circular representation of the pseudomolecule. Tracks II–V are repetitive sequence density (bin = 1 Mb), gene density (bin = 1 Mb), GC content (bin = 1 Mb), and genome collinearity, respectively. **b** Distribution of single-copy, multiple-copy, unique, other orthologous and unclustered genes in the 17 plant species used in this study: *Oryza sativa*, *Vitis vinifera*, *Pisum sativum, Phaseolus vulgaris*, *Vigna unguiculata*, *Vigna angularis*, *Glycine max, Pueraria lobata*, *Malus domestica*, *Cucumis melo*, *Momordica charantia*, *Populus alba*, *Salix brachista*, *Arabidopsis thaliana*, *Corchorus capsularis*, *Theobroma cacao*, and *Solanum lycopersicum*. **c** Number of overlapped gene families among the six legume species visualized in the UpSet plot. PSAT, *P. sativum*; VANG, *V. angularis*, PVUL, *P. vulgaris*, GMAX, *G. max*; PTHO, *P. thomsonii*; VUNG, *V. unguiculata*.

The pharmaceutical values of *P. lobata* and *P. thomsonii* are mostly attributed to their isoflavones, specifically daidzein and its glycosylated derivatives, daidzin and puerarin [1, 3, 10]. Puerarin is the chemotaxonomic marker and the major bioactive compound isolated from the roots of *Podalirius thomsonii*. The isoflavones and other metabolic compounds differ among *Pueraria* species, resulting in the distinct therapeutic characters of *Pueraria* species [11, 12]. Previous studies identified possible enzymes involved in the isoflavone biosynthesis pathway by comparing gene expressions or metabolite contents in different tissues of *P. lobata* [13–16]. Due to the complications attributable to the historical genome duplication as well as gene family expansions and contractions [8], little is known about the genes involved in isoflavone biosynthesis. A chromosome-level genome assembly with detailed function annotations could pave the way to elucidating the key players in the isoflavone biosynthesis pathway.

In this study, we generated a high-quality chromosome-level genome assembly of *P. thomsonii*, the first genome assembly in the *Pueraria* genus, by combining short and long-read DNA sequencing and Hi-C scaffolding technologies. We structurally and functionally annotated the genome with clustered gene families related to diverse biological processes. Comparative genomics analysis supported the idea that *P. thomsonii* is most closely related to soybean and experienced two historical whole-genome duplication (WGD) events. The key gene families and functional gene members in the isoflavonone and puerarin biosynthesis pathways, especially the UDP-glycosyltransferase gene family, were characterized based on a combination of genomic, transcriptomic, and metabolic data. The chromosome-level assembly contributes a new genome to the legume family, and lays a solid foundation for the genomic utilization of *P. thomsonii* both for legume science and genetic improvement of medicinal germplasm resources.

## Results

### Genome assembly and annotation

We firstly generated 78.95 Gb of 250 bp paired-end Illumina sequences. Genome complexity estimation based on *K*-mer analysis revealed that the diploid genome of *P. thomsonii* is ~1.35 Gb in size and has around 0.8% heterozygosity (Supplementary Fig. 1). We then *de novo* produced 91.6 Gb PacBio single-molecule long polymerase reads and assembled the genome using Falcon followed by assembly polishing using both the long and short reads. The total length of the PacBio assembly is 1.38 Gb with contig N50 of 598 kb. The assembly was further scaffolded into 281 scaffolds using Hi-C technology (Supplementary Fig. 2), 11 of which were at the chromosome level, accounting for 99.3% of the total assembly size (Fig. 1a, Supplementary Table 1). BUSCO analysis found that 92.9% of the core eukaryotic gene set were complete in our assembly, the majority of them being single-copy genes (Supplementary Table 3). These results suggest that the quality of the genome assembly is relatively high in contiguity and completeness.

The predicted repeat regions account for 62.70% of the genome assembly. Long terminal repeats (LTRs) were the main type of repetitive element, accounting for ~14.22% of the repeat regions. DNA transposons and long interspersed nuclear elements (LINEs) accounted for 4.03% and 2.47% of the assembly, respectively (Supplementary Table 3).

Structural annotation of the genome assembly yielded 45 270 gene models. On average, the gene length is 2446 bp, with the length of coding sequences 933 bp and an average of four exons per gene. Out of all gene models, 42 735 (~94.4% of the predicted protein-coding genes of *P. thomsonii*) were functionally annotated with known genes, conserved domains, or gene ontology (GO) terms (Supplementary Table  4, Supplementary File).

### Identification and evolution of gene families

We identified orthologs using the whole-genome gene sets of *P. thomsonii* and 16 other representative plant species, including five other species of the legume family (*Glycine max*, *Vigna angularis*, *Pisum sativum*, *Phaseolus vulgaris*, and *Vigna unguiculata*), 10 non-leguminous dicots (*Salix brachista*, *Corchorus capsularis*, *Arabidopsis thaliana*, *Cucumis melo*, *Malus domestica*, *Momordica charantia*, *Populus alba*, *Solanum lycopersicum*, *Vitis vinifera*, and *Theobroma cacao*), and one monocot species (*Oryza sativa*) as outgroup. In the *P. thomsonii* genome, 39 270 genes were clustered into 16 546 ortholog groups (Fig. 1b). The six legume species shared a core set of 11 204 gene families (Fig. 1c). Compared with *G. max*, 4743 gene families were expanded and 3099 gene families were contracted in *P. thomsonii* (Supplementary Fig. 3). These expanded gene families are functionally diverse and are functionally enriched in pathways such as flavonoids, alkaloids, sterols, and terpenoid biosynthesis (Supplementary Fig. 4). A total of 2373 gene families were unique in *P. thomsonii* in comparison with other leguminous species. These genes were enriched in functions related to nitrogen metabolism, streptomycin biosynthesis, ubiquinone, and other terpenoid-quinone biosynthesis (Supplementary Fig. 5).

Positive selection scanning of the 201 single-copy orthologous genes found 34 significant genes. Enrichment analysis of the functions of these positively selected genes indicated that they were enriched in functions such as circadian rhythm, homologous recombination, and starch and sucrose metabolism (Supplementary Fig. 6).

### Whole-genome duplication

**Figure 2:**
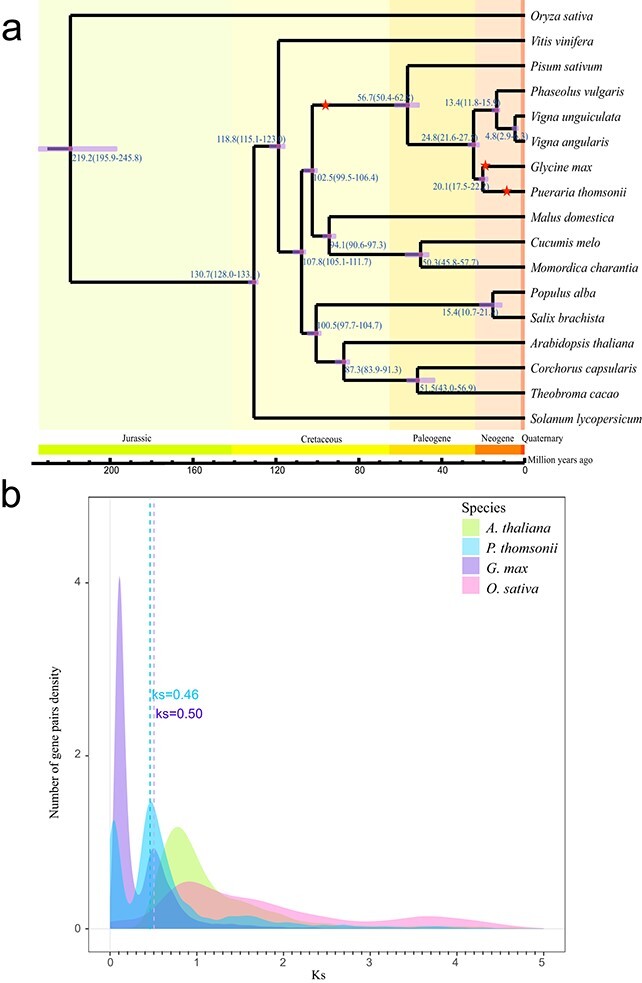
Evolution and WGD in *P. thomsonii* and 16 other species. **a** Phylogenetic trees and divergence times of the 17 species. The red stars on the branches represent occurrences of WGD events. **b** Distribution of *K*s values of *A. thaliana*, *P. thomsonii*, *G. max* and *O. sativa*.

A total of 201 single-copy orthologous genes shared across the 17 species were used to construct the phylogenetic tree. *P. thomsonii* was most closely related to *G. max*, giving an estimate of the divergence from soybean at about 20.1 million years ago (MYA), after the separation of Leguminosae and Orchidaceae at ~102.5 MYA (Fig. 2a). Both *K*s and 4-fold debatable third-codon transversion (4DTv) values were calculated based on homologous gene pairs in *P. vulgaris* and three other representative plant species, *G. max, A. thaliana*, and *O. sativa*. The distribution of *K*s or 4DTv values in *P. thomsonii* showed two peaks at *K*s values of around 0.05 (4DTv ~0.04) and 0.46 (4DTv ~0.2; Fig. 2b, Supplementary Fig. 7), which is consistent with published research findings [17, 18] that the *K*s range is between 0.4 and 1.2. Calculated from the *r* value of 5.17 × 10^−3^ in published studies [19], the first peak occurred after the divergence of *P. thomsonii* from *G. max* and was dated at ~4.8 Mya, later than the onset of the most recent WGD in *G. max*. The second peak, shared between *P. thomsonii* and *G. max*, resulted from a much older WGD, which happened in the common ancestor of the legume species, at ~44.5 Mya.

### Combined metabolic and gene expression analyses reveal isoflavone biosynthesis pathway

**Figure 3:**
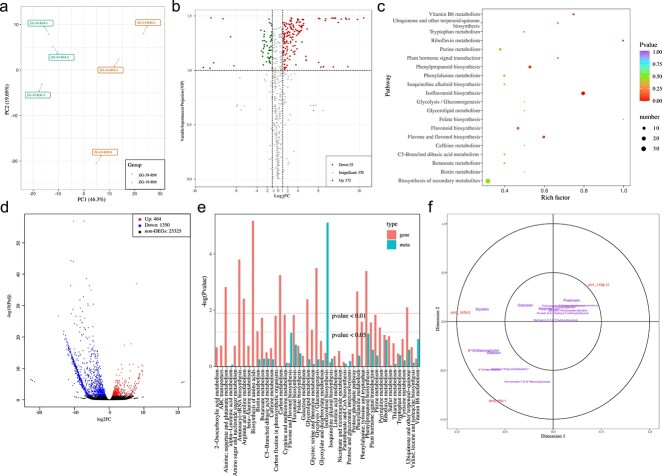
General overview of metabolome and transcriptome. **a** PCA of metabolites. **b** Volcano plot of differential metabolites between the roots of ZG-19 and ZG-39. **c** KEGG enrichment analysis of the DMs between the roots of ZG-19 and ZG-39. **d** Volcano plot of DEGs between the roots of ZG-19 and ZG-39. **e** KEGG enrichment pathway with both DMs and DEGs. The red bars are the negative log_10_ and the green color represents the enrichment *P*-value of DMs, which is expressed as −log(*P*-value), and higher vertical coordinates represent stronger enrichment. **f** Canonical correlation analysis between the DMs and DEGs in the isoflavonoid biosynthesis pathway. Gene IDs are in green and metabolite names are in purple; within the same region, the further from the origin and the closer to each other the higher the correlation.

We performed untargeted metabolic profiling using the roots of high- and low-puerarin genotypes, ZG-19 and ZG-39, respectively. A total of 614 metabolites were detected. Both principal component analysis (PCA) and correlation analysis showed that biological repeat samples are much more closely related within genotypes than between genotypes (Fig. 3a, Supplementary Fig. 8). A total of 225 differential metabolites (DMs) were identified between the two genotypes, of which 172 were up-regulated and 53 were down-regulated in ZG19 relative to ZG-39 (Fig. 3b, Supplementary File ). GO enrichment analysis of the DMs showed significant differences in the synthetic pathways of flavonoids, flavonol, and isoflavones between ZG19 and ZG-39 (Fig. 3c).

A total of 1814 differentially expressed genes (DEGs) were identified between the two genotypes, of which 464 were upregulated and 1350 were downregulated in ZG19 relative to ZG-39 (Fig. 3d). The enriched function categories of the DMs and DEGs overlap; the results showed that they were all genes or metabolites relevant to flavonoids, isoflavones, and ATP-binding cassette transport (Fig. 3e). We identified a large number of metabolites and gene pairs that were highly correlated across the samples with |*r*| > 0.8 (Supplementary Figs. 9 and 10). Pairwise correlation coefficients were calculated between the identified metabolites and genes. Sixty percent of all the significant correlations involved upregulated metabolites and downregulated or unchanged genes. In 15% of the significant correlations, metabolites and gene expressions changed in the same direction (Supplementary Fig. 9). Moreover, we identified a large number of DEGs and DMs in the isoflavone biosynthesis pathway (Supplementary Fig. 11). Canonical correlation analysis of the DEGs and DMs in the isoflavone biosynthesis pathway showed that the expression of gene chr11g3_854.1, encoding 2-hydroxyisoflavanone synthase, was highly correlated with the content of daidzein in the tubers, while the expression of chr11g1_1158.17, which encodes UDP-glycosyltransferase, was highly correlated with the pratensein content in the tubers (Fig. 3f).

### Puerarin synthesis pathway

**Figure 4:**
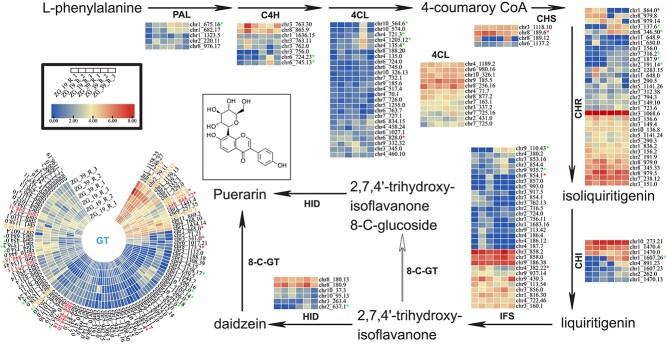
Expression profile of genes encoding enzymes of the puerarin biosynthesis pathway in the roots of ZG-19 and ZG-39 genotypes. Gene expression values (FPKM) were scaled by log_2_(FPKM+1). Red and blue asterisks represent significant up- and downregulation of gene expression in root tissue of ZG-19 relative to ZG-39, respectively. Expression profile of all members of the glucosyltransferase (*GT*) gene family in *P. lobata* var. *thomsonii* are presented; genes in red and orange are homologous to *8-C-GT* genes. Moreover, genes in red are the homologous genes of *PIUGT43*, which was previously found to catalyze the C-glucosylation of daidzein to puerarin [16]. PAL, phenylalanine ammonialyase; C4H, cinnamate-4-hydroxylase; 4CL, 4-coumarate-CoA ligase; CHS, chalcone synthase; CHR, chalcone reductase; CHI, chalcone isomerase; IFS, 2-hydroxyisoflavanonesynthase; 8-C-GT, 8-C-glucosyltransferase; HID, 2-hydroxyisoflavanone dehydratase.

We identified 572 genes homologous to nine gene families putatively encoding the enzymes in the puerarin synthesis pathway (Supplementary File 3). All nine gene families in the *P. thomsonii* genome were expanded compared with the other five legume species (Supplementary Fig. 12). We predicted that out of all the homologous genes, 40 genes differentially expressed between the high- and low-puerarin roots were functionally involved in the puerarin biosynthesis pathway in *P. thomsonii* (Fig. 4, Supplementary File 4). We further analyzed the genes in the glycosyltransferase family catalyzing the glycosylation modification. A total of 104 glucosyltransferase (*GT*) genes were identified in *P. thomsonii*. There were 13 genes homologous to 8-C-glucosyltransferase (*8-C-GT*), 6 of which were homologous to the previously studied *PIUGT43* gene catalyzing the C-glucosylation of daidzein to puerarin [14]. The DEGs in the *GT* family with the top three largest gene expression changes in the roots of ZG-19 and ZG-39 were chr11g8_848.22, chr11g8_844.18, and chr11g7_666.8. We also identified 179 genes significantly correlated with the puerarin content in the roots (Supplementary File 5).

The gene (chr11g3_854.1) encoding isoflavone synthase, which catalyzes the synthesis of daidzein, was identified to be highly associated with the synthesis pathway of puerarin. Moreover, daidzein was detected as an intermediate metabolite in puerarin synthesis, implying that gene chr11g3_854.1 likely promotes daidzein synthesis and accumulates in the roots. The synthesis of puerarin is also likely to be dependent on daidzein via glycosyltransferase chr11g8_848.22 or chr11g8_844.18.

## Discussion

*Pueraria* has great ecological and economical impact globally, yet genomic resources and understanding are still limited at the level of molecular markers and transcriptomes. Our study provided a foundational resource by providing the first high-quality, chromosome-level genome of *P. thomsonii* by combining long-read sequencing and Hi-C scaffolding technologies*.* The genome sizes estimated using short reads and from the assembly were very close, at 1.35–1.38 Gb, which is relatively large, with a high percentage of repeat regions compared with other legume species.

We found that *P. thomsonii* was most closely related to soybean based on genomic phylogeny; both went through a shared ancient WGD as well as a lineage-specific WGD. Large genomic blocks of *G. max* were found in synteny with much more fragmented chromosome regions in *P. thomsonii* (Supplementary Fig. 13). This suggested that the *P. thomsonii* genome had undergone extensive chromosome rearrangement despite its estimated more recent WGD compared with *G. max*, which coincided with the smaller number of chromosomes in *P. thomsonii* [15].

Analyzing the annotated gene set revealed that, compared with five other leguminous species, *P. thomsonii* is unique in that ~14.3% of the gene families were enriched in pathways related to sugar and starch metabolism (Supplementary File 6), which coincided with the characteristically high starch and sugar content in *P. thomsonii* roots. These unique genes could shed light on the genomics of starch accumulation in root tissues in the legume family.

Puerarin is the most abundant bioactive compound in the *Pueraria* genus. Although the genes and enzymes involved in the puerarin synthesis pathway have been studied [13, 16, 20], our study for the first time identified and characterized the genes of the puerarin biosynthesis pathway using multi-omic data collected at the genomic, transcriptomic, and metabolic levels. The results showed that all gene families in the puerarin biosynthesis pathway were expanded compared with other legume species and 40 out of all 235 homologous genes were functionally involved in the pathway.

Moreover, we analyzed the *GT* gene family, encoding a key enzyme that catalyzes glycosylation modifications at different stages downstream of flavone or isoflavone biosynthesis and confers biological and pharmochemical properties in isoflavonoids such as puerarin. We constructed a phylogenetic tree using the 104 glucosyltransferase genes filtered and identified in this study along with previously characterized UGTs. We then classified these genes into seven clusters/subfamilies according to their classification in the previous study [16] (Supplementary Table 5, Supplementary Fig. 14). There were six genes in *P. thomsonii* that belonged to cluster 6, characterized as a plant isoflavonoid C-glycosyltransferase (*CGT*) group. However, our phylogeny analysis of the *UGT* genes placed the six genes as well as their homologous genes from *P. lobata* within the (iso)flavone 7-OH glycosylation group (cluster 5), which was inconsistent with the previous classification, and suggested further verification was needed.

The reference genome of *P. thomsonii* not only reveals the genes of the puerarin synthesis pathway more comprehensively, but also provides the possibility of gene localization, screening and evolutionary studies. This is very important for the cultivation of *P. thomsonii* as a herbal medicine.

### Conclusions

We report the first high-quality chromosome-scale genome of *P. thomsonii*. The genome size is ~1.37 GB, with contig N50 of 593.70 kb. Approximately 99% of the assembled sequences were represented by 11 pseudochromosomes. In total, we identified ~62.70% of the repetitive sequences and 45 270 protein-coding genes. The *P. thomsonii* genome experienced two WGD events, the first at ~44.5 Mya and the most recent one at ~4.8 Mya after divergence from *G. max*. Using multi-omics data, genes and metabolites were found associated with puerarin content in root tissues and were enriched in isoflavone biosynthesis and sugar and starch metabolic pathways. Nine gene families involved in the puerarin biosynthesis pathway were characterized, and 235 candidate genes and 13 key glycosyltransferase candidate genes were further selected. Our study developed the important multi-omic resources for exploiting and improving *P. thomsonii* as an economic crop for edible starch and traditional Chinese medicine.

## Materials and methods

### Genome sequencing

For genome sequencing, we grew and labeled plants of *P. thomsonii* from the Lijian Scientific Research Base of Guangxi Academy of Agricultural Sciences (GXAAS). Fresh, young, and healthy leaves were harvested from well-growing individuals, and were immediately frozen in liquid nitrogen and stored at −80°C for DNA extraction. High-quality genomic DNA was extracted from the harvested samples using a modified cetyltrimethyl ammonium bromide (CTAB) method [21]. For third-generation genome sequencing, the SMRT Bell library was prepared using SMRTbell Express Template Prep Kit 2.0, which was loaded into a Pacific Biosciences PacBios loaded into a Prep Kit 2.0 Sequel II instrument on PacBio SMRT cells 8 M (Pacific Biosciences, Menlo Park, CA, USA), acquiring one movie of 30 hours per SMRT cell. For short-read sequencing, the Illumina library with average insert sizes of 397 bp was generated using a Genomic DNA Sample Preparation Kit (Illumina), which was loaded into an Illumina 6000 instrument with PE250.

### Hi-C sequencing

To construct chromosome-level superscaffolds, Hi-C sequencing data were produced as previously described [22]. Briefly, fresh samples were cut into small segments and cross-linked by immersion in 3% formaldehyde for 15 min. Then the material was crushed to a fine powder, which was used to isolate the nuclei. The isolated nuclei were processed by purifying, digesting with MboI, blunt-end repairing, and labeling with biotin. After processing, the DNA was re-ligated with T4 DNA ligase. After digestion with proteinase K and cross-linking reduction using formaldehyde, biotin-containing DNA fragments were captured and used to construct Hi-C libraries. We obtained 127.5 Gb of sequencing data by sequencing the final library using the Illumina X Ten sequencing platform.

### Genome survey

Short reads produced by the Illumina 6000 platform were quality-filtered by HTQC [23] (version v1.92.310) using the following method. Adaptors were firstly removed from the sequencing reads, and read pairs with any one end having an average quality <20 were discarded. Ends of reads were trimmed if average quality was <20 in the sliding window size of 5 bp, and read pairs with any end shorter than 75 bp were removed. The quality-filtered reads were used for estimation of genome size. The 17-mer occurrence distribution of sequencing reads was generated from short libraries with Jellyfish [24] (v2).

### Genome assembly

Subreads generated with the above long-read third-generation sequencing were used for genome assembly of *P. lobata*. The draft assembly of the genome was made using Falcon (v0.3.0) [25]. To correct errors in the primary assembly, Racon (v1.44) tools were used to polish the genome [26]. Illumina-derived short reads were used to correct any remaining errors by NextPolish (v1.1.0) [27].

To scaffold the contigs, 425 011 698 clean-read pairs were sequenced from the Hi-C library. Fastp (0.19.5) [28] was used to filter Hi-C data, including removing adaptors and low-quality reads. HiC-Pro Proto was used to obtain effective reads [29]. After filtering, 6% of high-quality and effective Hi-C data was retained. The resulting effective reads were mapped to the assembled and polished *P. thomsonii* genome using BWA (bwa-0.7.17) [30] with default parameters. ALL-HiC software was used to anchor scaffolds to chromosomes to obtain the chromosome-level *P. thomsonii* assembly.

### Genome structural and functional annotation

We used LTR_finder (v1.07) [31], repeatscont (v1.0.5) [32], and trf (v4.09) [33] tools to identify repetitive elements in the *P. thomsonii* genome assembly, then used these sequences as the library, and finally used RepeatMasker (open-4.0.9) [34] to conduct repeat annotation. Maker (v3.01.02) [35] was used to annotate gene structures, using the protein sets of *Medicago sativa, Lotus japonicus*, and *G. max* retrieved from the NCBI database as homology evidence.

Gene functions were inferred according to the best match of alignments to NCBI Swiss-Prot [36] protein databases using BLASTP (NCBI BLAST v2.6.0+) [37, 38] and the KEGG database [39] with an e-value threshold of 1e−5. GO [40] IDs were obtained by using Blast2GO [41].

### Gene family identification

To identify ortholog groups of the protein-coding genes, whole-genome protein sequences were pairwise aligned using BLASTP programs (NCBI BLAST+ v2.6.0) with a maximal e-value of 1e−5 from *P. thomsonii* and other 16 species, including *O. sativa*, *G. max*, *S. brachista*, *C. capsularis*, *A. thaliana*, *C. melo*, *M. domestica*, *M. charantia*, *P. vulgaris*, *P. alba*, *S. lycopersicum*, *V. angularis*, *V. vinifera*, *P. sativum*, *T. cacao*, and *V. unguiculata*. To exclude putatively fragmented genes, identity <30%, coverage <50% and g protein-encoding sequences shorter than 50 bp amino acids were filtered out. OrthoMCL (v14–137) [42] was used to cluster genes into ortholog groups with the parameter of inflation set as 1.5.

### Phylogenetic analysis

Single-copy orthologs were used to construct a phylogenetic tree for *P. thomsonii* and the other 16 species. Protein sequences were aligned with MUSCLE (v3.8.31) [43], and alignments of the corresponding coding sequences were generated and concatenated with the guidance of protein alignment. The maximum likelihood method was used to construct the phylogenetic tree with RAxML (v8.2.11) [44].

### Gene family expansion and contraction analysis

Based on the identified gene families and the phylogenetic tree with predicted divergence times of these species, CAFÉ [45] was used to analyze gene family expansion and contraction, by using a random birth and death model. A conditional *P*-value was calculated for each gene family, and families with a conditional *P*-value <.05 were considered to have accelerated rates of gene gain or loss. The significantly expanded and contracted gene families in *P. thomsonii* were mapped to KEGG pathways for enrichment analysis, using a hypergeometric test with false discovery rate (FDR)-adjusted *P*-value (*Q*-value) <0.05 (https://github.com/StoreyLab/qvalue).

### Positively selected genes

Based on the phylogenetic tree, the rate ratio (*ω*) of non-synonymous (*K*a) to synonymous (*K*s) nucleotide substitutions was estimated using the PAML (v4.9e) package [46] to scan the selective constraints on the candidate genes that were single-copy across all 17 species. After high-quality alignments of related sequences were obtained as described above, we compared a series of evolutionary models in the likelihood framework using the species trees. A branch-site model was used to estimate average *ω* across the tree (*ω*0), *ω* of the appointed branch to test (*ω*2), and *ω* of all other branches (*ω*1). Genes under positive selection were identified when the adjusted *P*-value was <.05.

### Whole-genome duplication analysis

We used the synonymous substitution rate (*K*s) to detect WGD events. First, gene pairs used in the *K*s and 4DTv calculations were acquired using JCVI [47] between *A. thaliana*, *O. sativa*, *G. max*, and *P. thomsonii*. Specific methodology was consistent with previous studies [48]. *K*s values were calculated using KaKs_Calculator [49] (v2.0). Finally, the *K*s distribution was used to evaluate WGD events. WGD events were dated using the local multi-rate clock and *t* = *K*s/2*r* in which *r* = 5.17 × 10^−3^ refers to the nucleotide substitution rate [19].

### RNA sequencing and expression analysis

We chose two genotypes for RNA-Seq: ZG-19 with high puerarin content and ZG-39 with low puerarin content. Freeze-dried root samples were collected and immediately frozen in liquid nitrogen. For each genotype, three biological replicates were collected in parallel. RNA was isolated using the Plant RNA Kit (R6827, Omega), and first-strand cDNA was synthesized using random primers with TransScript II All-in-One First-Strand cDNA Synthesis SuperMix for qPCR (One-Step gDNA Removal) (AT341, Transgen Biotech). One microgram of RNA per sample was used as input material for RNA-Seq. RNA-Seq libraries were prepared using the Illumina mRNA-Seq Library Preparation Kit and sequenced using an Illumina HiSeq 2000 with 150 bp paired-end read sequencing.

Low-quality reads, such as adaptors, reads containing >5% unknown nucleotides, and reads with Q20 <20% (percentage of sequences with sequencing error rates <1%), were removed by fastqc. The clean reads were mapped to *P. thomsonii* reference genome using HISAT2 [50]. We predicted new transcripts from the genome alignment using StringTie [51] and mapped the reads to the merged transcriptome set using Bowtie2 [52] and quantified the normalized expression level [reads per kilobase of transcript per million reads mapped (FPKM)] of each gene and transcript using RSEM [53]. DESeq2 [54] (version 1.22.2) was used to identify differentially expressed genes (DEGs) with |log2(fold change)| ≥1 and the adjusted FDR <0.05. DEGs were functionally annotated using the KEGG database. Enrichment analysis was performed for annotated DEGs.

### Untargeted metabolic profiling

In addition to RNA extraction samples, another set of six samples from ZG-39 and ZG-19 were collected for metabolic profiling. They were freeze-dried and crushed using a mixer mill (MM 400, Retsch) with a zirconia bead for 1.5 min at 30 Hz. For each sample, 100 mg powder was weighed and extracted overnight at 4°C with 1.2 ml 70% aqueous methanol. Following centrifugation at 12 000 rpm for 10 min, the extracts were filtered with an SCAA-104 (0.22 μm pore size; Anpel, Shanghai, China, http://www.anpel.com.cn/) before UPLC–MS/MS analysis.

The extracts were analyzed using an UPLC–ESI–MS/MS system (UPLC, Shimadzu Nexera X2, www.shimadzu.com.cn/; MS, Applied Biosystems 4500 Q TRAP, sciex.com). Except for the Analyst v1.6.3 software and ion spray voltage 5500 V (positive ion mode)/−4500 V (negative ion mode), other HPLC conditions, linear ion trap and triple quadrupole (QQQ) scans and experiments were the same as previously described [55].

### Differential metabolic analysis

PCA was performed by the function prcomp in R (www.r-project.org) before scaling the data by unit variance [56]. Results for samples and metabolites [hierarchical cluster analysis (HCA)] are presented as heat maps and dendrograms, while Pearson’s correlation coefficient (PCC) between samples was calculated by the cor function in R and presented as heat maps only. Both HCA and PCC were conducted using the R package pheatmap [57]. Metabolites differentially regulated between genotypes were defined by the variable importance in projection (VIP) > = 1 and |log2(fold change)| ≥ 1. VIP values were extracted from the orthogonal partial least squares discrimination analysis (OPLS-DA) results, which also contained score maps and permutation maps, generated using the R package MetaboAnalystR [58]. The data were log-transformed (log_2_) and mean-centered before OPLS-DA analysis. A permutation test (200 permutations) was carried out to avoid overfitting. Identified metabolites were annotated using the KEGG compound database (http://www.kegg.jp/kegg/compound/), then mapped to the KEGG pathway database (http://www.kegg.jp/kegg/pathway.html). The mapped pathways with significantly regulated metabolites were fed into metabolite sets enrichment analysis (MSEA) and significance was determined by the *P*-value of the hypergeometric test.

### Comparison between differential metabolites and differentially expressed genes

PCCs were calculated between DMs and DEGs across the six samples. The DMs and DEGs from comparison pairs with PCC >0.8 or <−0.8 were retrieved for downstream analysis. Pairwise PCC analysis was performed on the kept metabolites and genes and plotted in a heatmap to visualize their correlation patterns.

### Identification of genes in puerarin biosynthesis pathways

Sequences of a list of known gene families encoding enzymes that are involved in puerarin biosynthesis were aligned against the genome-wide gene set of *P. thomsonii* using BLASTP with e-value ≤1e−5 and identity ≥40%. The key structural domains from pfam [59] and InterPro [60] of the enzymes were queried by HMMER [61] to filter for the bona fide genes in *P. thomsonii*. Genes possibly involved in puerarin biosynthesis, including the chalcone isomerase (CHI), phenylalanine ammonialyase (PAL), cinnamate-4-hydroxylase (C4H), 4-coumarate-CoA ligase (4CL), chalcone synthase (CHS), chalcone reductase (CHR), 2-hydroxyisoflavanone synthase (IFS/2-HIS), glucosyltransferase (GT), and 2-hydroxyisoflavanone dehydratase (HID), were identified by the same approach in the *P. thomsonii* genome.

## Acknowledgements

This work was supported by the National Natural Science Foundation of China (31960420, 31870275), the Guangxi Key R&D Program Project (Guike AB1850028), the Guangxi Natural Science Foundation Project (2018GXNSFBA294001, 2019GXNSFBA245093), and the Special Project for Basic Scientific Research of Guangxi Academy of Agricultural Sciences (Guinongke 2021YT057). We thank Dr Shiyu Chen and Prof. Qiusheng Kong for proofreading the manuscript.

## Author contributions

X.H.S., X.X.Y., and H.B.Y. conceived and designed the study. K.P.O, W.D.Z., S.C., and L.Y.L. contributed to sample preparation. L.X., Y.S.Z., D.H., Z.B.X., R.H.M., and D.Q.W. participated in data analysis and substantively revised the manuscript. All authors read and approved the final manuscript.

## Data availability

This Whole Genome Shotgun project and Transcription project of *P. lobata* var. *thomsonii* has been deposited in DDBJ/ENA/GenBank and SRA under the umbrella of BioProject Accession PRJNA723378. The genome accession described in this paper is JAGTWZ000000000.

## Conflict of interest

The authors declare that they have no conflict of interest.

## Supplementary data

Supplementary data is available at *Horticulture Research Journal* online.

## Supplementary Material

Web_Material_uhab035
